# Putrescine treatment reverses α-tocopherol-induced desynchronization of polyamine and retinoid metabolism during rat liver regeneration

**DOI:** 10.1186/s12967-016-1062-y

**Published:** 2016-10-26

**Authors:** Lourdes Sánchez-Sevilla, Edgar Mendieta-Condado, Rolando Hernández-Muñoz

**Affiliations:** Departamento de Biología Celular y Desarrollo, Instituto de Fisiología Celular, Universidad Nacional Autónoma de México (UNAM), Apdo. Postal 70-243, 04510 Mexico City, DF Mexico

**Keywords:** Cell proliferation, Ornithine decarboxylase, Putrescine, Lipid peroxidation, Citrulline

## Abstract

**Background:**

The pre-treatment with α-tocopherol inhibits progression of rat liver proliferation induced by partial hepatectomy (PH), by decreasing and/or desynchronizing cyclin D1 expression and activation into the nucleus, activation and nuclear translocation of STAT-1 and -3 proteins and altering retinoid metabolism. Interactions between retinoic acid and polyamines have been reported in the PH-induced rat liver regeneration. Therefore, we evaluated the effect of low dosage of α-tocopherol on PH-induced changes in polyamine metabolism.

**Methods:**

This study evaluated the participation of polyamine synthesis and metabolism during α-tocopherol-induced inhibition of rat liver regeneration. In PH-rats (Wistar) treated with α-tocopherol and putrescine, parameters indicative of cell proliferation, lipid peroxidation, ornithine decarboxylase expression (ODC), and polyamine levels, were determined.

**Results:**

Pre-treatment with α-tocopherol to PH-animals exerted an antioxidant effect, shifting earlier the increased ODC activity and expression, temporally affecting polyamine synthesis and ornithine metabolism. Whereas administration of putrescine induced minor changes in PH-rats, the concomitant treatment actually counteracted most of adverse actions exerted by α-tocopherol on the remnant liver, restituting its proliferative potential, without changing its antioxidant effect. Putrescine administration to these rats was also associated with lower ODC expression and activity in the proliferating liver, but the temporally shifting in the amount of liver polyamines induced by α-tocopherol, was also “synchronized” by the putrescine administration. The latter is supported by the fact that a close relationship was observed between fluctuations of polyamines and retinoids.

**Conclusions:**

Putrescine counteracted most adverse actions exerted by α-tocopherol on rat liver regeneration, restoring liver proliferative potential and restituting the decreased retinoid levels induced by α-tocopherol. Therefore interactions between polyamines and retinol, mediated by the oxidant status, should be taken into consideration in the development of new therapeutic strategies for pathologies occurring with liver cell proliferation.

## Background

The α-tocopherol is the predominant form of vitamin E in the human plasma, and the most effective antioxidant tocopherol (α > β > γ > δ) [1]; however, γ-tocopherol, but not α-tocopherol, has anti-inflammatory properties [2]. As to the effects of α-tocopherol in the diseased liver, efforts have been made to evaluate the impact of vitamin E (VE) on hepatocellular carcinoma (for review, see Hernández-Muñoz et al., Ref. [3]). Several vitamins such as retinoic acid, ascorbic acid, vitamin D and E are known to prevent the development and progression of breast cancer [4]; indeed, retinoic acid and α-tocopherol act synergistically in inhibiting human breast cancer cell proliferation, upregulating antioxidant enzymes and proteins involved in apoptosis [5]. Moreover, VE plays a protective effect against cigarette smoke extract-induced cytotoxicity in mouse embryonic lung cells, apparently involving the mitochondrial pathway of cytochrome c-mediated caspase activation [6]. In the same context, abdominal obesity is a risk factor associated with enhanced oxidative stress; it has proved that this condition has relationships with dietary vitamin E and A intake and genetic variants of thioredoxin and catechol-*O*-methyltransferase [7]. In this regard, we have demonstrated that the oxidant status can control the progression of partial hepatectomy (PH)-induced rat liver regeneration [8, 9], and treatment with the α-tocopherol promotes an early termination of priming cell events, culminating in a partial inhibition of rat liver regeneration [10].

The proliferating liver after PH is highly sensible to small dosing of α-tocopherol, which alters the pattern of signal transducer and activator of transcription (STAT) protein activation, and blunts retinoic acid formation by decreasing alcohol dehydrogenase (ADH) activity [11, 12], probing that reactive oxygen species (ROS) participate in changing the cell redox state during liver cell proliferation [11]. Retinoic acid is synthesized in the liver and can interact with retinoid receptors which control expression of a large number of genes involved in hepatic processes [13]. We have demonstrated that interactions between α-tocopherol and retinoid compounds (retinol, retinal, and retinoic acid) are important for impacting rat liver regeneration after PH. In this context, an anti-tumor effect of VE might be attributed to a kind of disruption of signal transduction [14], as we have already explored [10, 15]. In this regard, it has been highlighted the role of STAT3 signaling in liver injury, steatosis, inflammation, regeneration, fibrosis, and hepato-carcinogenesis, proposing that cytokines and small molecules that activate STAT3 in hepatocytes may readily have therapeutic benefits to treat liver diseases, including cancer [16].

As well as retinoid metabolism, the polyamines are also required for animal cell proliferation, since activation of polyamine catabolism invariably leads to growth inhibition [17]. The PH-induced rat liver regeneration is closely linked to synthesis and metabolism of polyamines, and its inhibition resulted in decreased hepatic DNA synthesis [18], which is reversed by supplementing the polyamine putrescine [19]. Cellular polyamine concentrations are highly regulated, since enhanced levels of these molecules can dysregulate polyamine homeostasis leading to toxic cellular effects. In turn, low levels of polyamines can inhibit cell proliferation and affect embryo development [20]. In this context, perioperative oral polyamine administration attenuates liver ischemia-reperfusion injury and promotes liver regeneration [21].

Moreover, there is evidence pointing out interactions between retinoic acid and polyamines. The transglutaminase activity (or transamidation function) can cross-link polyamines to target proteins, and retinoic acid increases expression/activation of transglutaminase [22]. Depletion of transglutaminase activity by cadaverine enhances toxicity of retinoids [23], which can be counteracted by polyamines in several tissues [24].

Based on the aforementioned, we sought that the inhibitory effect of α-tocopherol on rat liver regeneration could be linked to altered ODC expression and polyamine synthesis and metabolism, in a similar fashion that involves disturbed ADH-mediated retinoid metabolism [12]. Therefore, it is likely that putrescine administration can partial or completely rescue the adverse effects of α-tocopherol on rat liver regeneration.

## Methods

### Animals and treatments

Male Wistar rats weighing 240–270 g (3 months old n = 120), were housed with free access to food and water. Animals were randomly divided into two groups: rats receiving a daily intragastric administration of 6 IU/kg of α-tocopherol (approximately 4 mg/kg of VE) diluted in peanut oil (1 mL/rat), and those receiving only the α-tocopherol vehicle [10]. After completing the treatment with α-tocopherol, animals were again divided according to their surgical status. The 70 % PH was performed according to the previously described [8] and sham-operated animals provided a surgical control. Another set of sham- and PH-rats, treated or not with α-tocopherol, received an intraperitoneally single dose of 40 mg/kg of body (0.3 mmol/kg of b.w.) of putrescine, as previously reported [19]. Afterwards, rats were euthanized under sodium pentobarbital anesthesia and liver samples were obtained. All manipulations were done according to our Institutional Guide for Animal Experimentation and Care (National University of Mexico).

### Liver histology and mitotic index

**Fig. 1:**
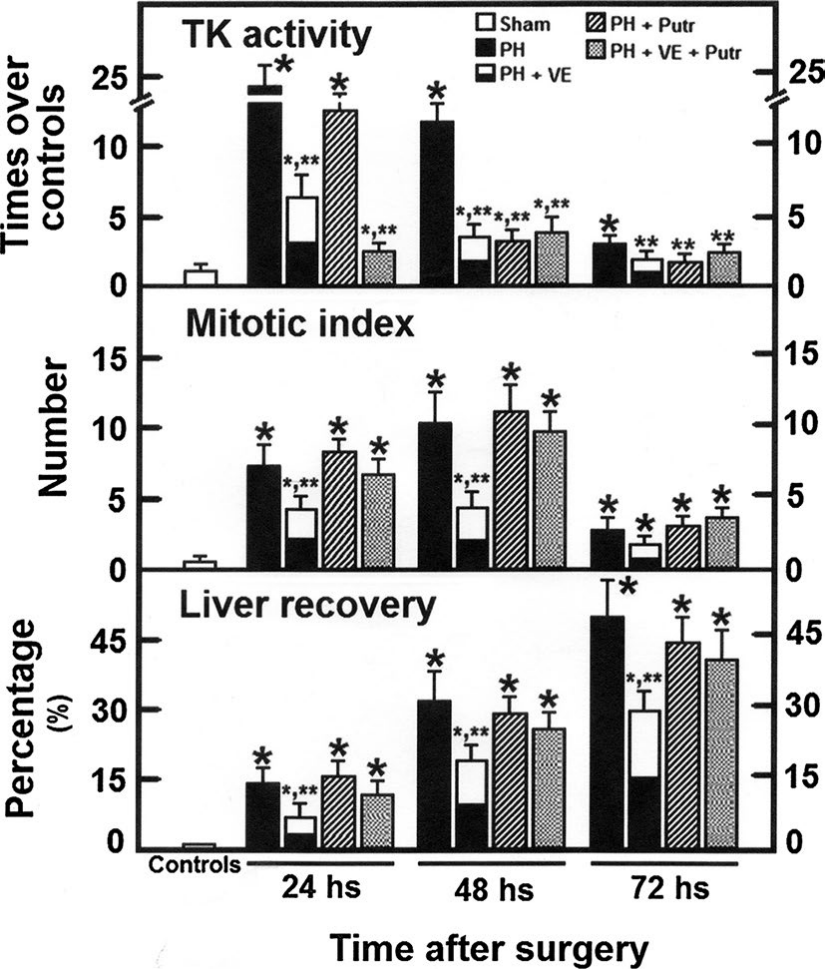
Parameters of liver cell proliferation in animals subjected to PH and treated with α-tocopherol and/or putrescine. Results are the mean ± SE of 5 individual observations per experimental group for cytosolic TK activity (controls = 0.20 nmol of [^3^H] TMP formed h^−1^ mg^−1^ of protein), for mitotic index (number of mitotic cells in 20 microscopic fields with a 40× objective), and those for the gain of liver mass (%) after PH. *Symbols* for each experimental group are indicated at the top of the figure. Statistical significance: *p < 0.01 against the control group, and **p < 0.01 vs. the group with PH only

Hepatic samples from each group (after 48 h of surgery) were used for light microscopy stained with hematoxylin-eosin. Evaluated criteria for the analysis of morphological abnormalities were the same as previously reported [25]: fatty infiltration, degree of inflammation, and hepatocellular disorganization. Mitotic index corresponded to the number of mitotic cells in 10 microscopic fields with a 40× objective, and expressed as number of mitosis per field (Fig. 1).

### Biochemical analyses

The cytosolic and plasma membrane fractions were obtained by differential centrifugation, as described by Aguilar-Delfín et al. [8]. The ROS levels in sub-cellular fractions were estimated through the method described by Viarengo et al. [26], using the fluorescence signal generated by ROS reacting with 2′,7′-dichlorodihydrofluorescein di-acetate (H2DCF-DA, Molecular Probes). The thymidine kinase (TK) activity was determined according to Sauer and Willmans [27]. The ODC activity was detected by using [1-^14^C] ornithine (specific activity, 54 mCi/mmol), according to Diehl et al. [19]. Liver levels for ornithine and citrulline were determined as previously described, in detail [28].

### Quantification of polyamines and retinoid levels by HPLC

Liver levels of polyamines (putrescine, spermidine, and spermine) present in acid-extracts from cytosol were measured as benzoyl derivatives using HPLC (Beckman-Golden system HPLC with UV detector), prepared essentially as described by Thyssen et al. [29]. The equipment used was a Beckman-Golden system HPLC with UV detector, and a C-18 reverse column was employed. For retinoids, total liver homogenate (200 mL) was extracted with 2 mL of methanol/acetone 1:1 v/v), suspended in methanol/dimethyl sulfoxide (1:1 v/v), and analyzed in the HPLC, as described in detail by Molotkov et al. [30].

### Western-blot analyses for cytosolic amount for ODC

Thirty five µg per well of cytosolic protein were separated in a 10 % SDS-PAGE through increasing voltage from 65 to 110 V. After separation, proteins were transferred to nitrocellulose membrane in a Tobwin buffer at 250 mA 2 h in 4 °C, blocking unspecific sites with PBS-Tween 0.3 % buffer (pH = 7.4) containing casein 1 % and gelatin 0.3 %, followed by an overnight incubation with primary antibody (0.02 µg/mL of monoclonal anti-ODC mouse antibody, SIGMA-Aldrich chemical CO.) and 60 min-incubation with a secondary antibody coupled to horseradish peroxidase (0.001 µg/mL, Santacruz, CA). Afterwards, membranes were incubated with the chemiluminescent kit (Millipore Inc.) and exposed to photographic film (Kodak Quimioluminicents Film). Data was analyzed through Sigma Stat Software 5.0.

### Statistical analysis

Results are expressed as mean ± SD, and statistical significance of the differences was assessed by two-way ANOVA for a normal distribution of data. In the case of significance, a Newman Keuls test was further applied and a p < 0.01 value was considered as significant.

## Results

### Liver histology and parameters indicative of liver cell proliferation in animals subjected to PH and treated with α-tocopherol and/or putrescine

**Fig. 2:**
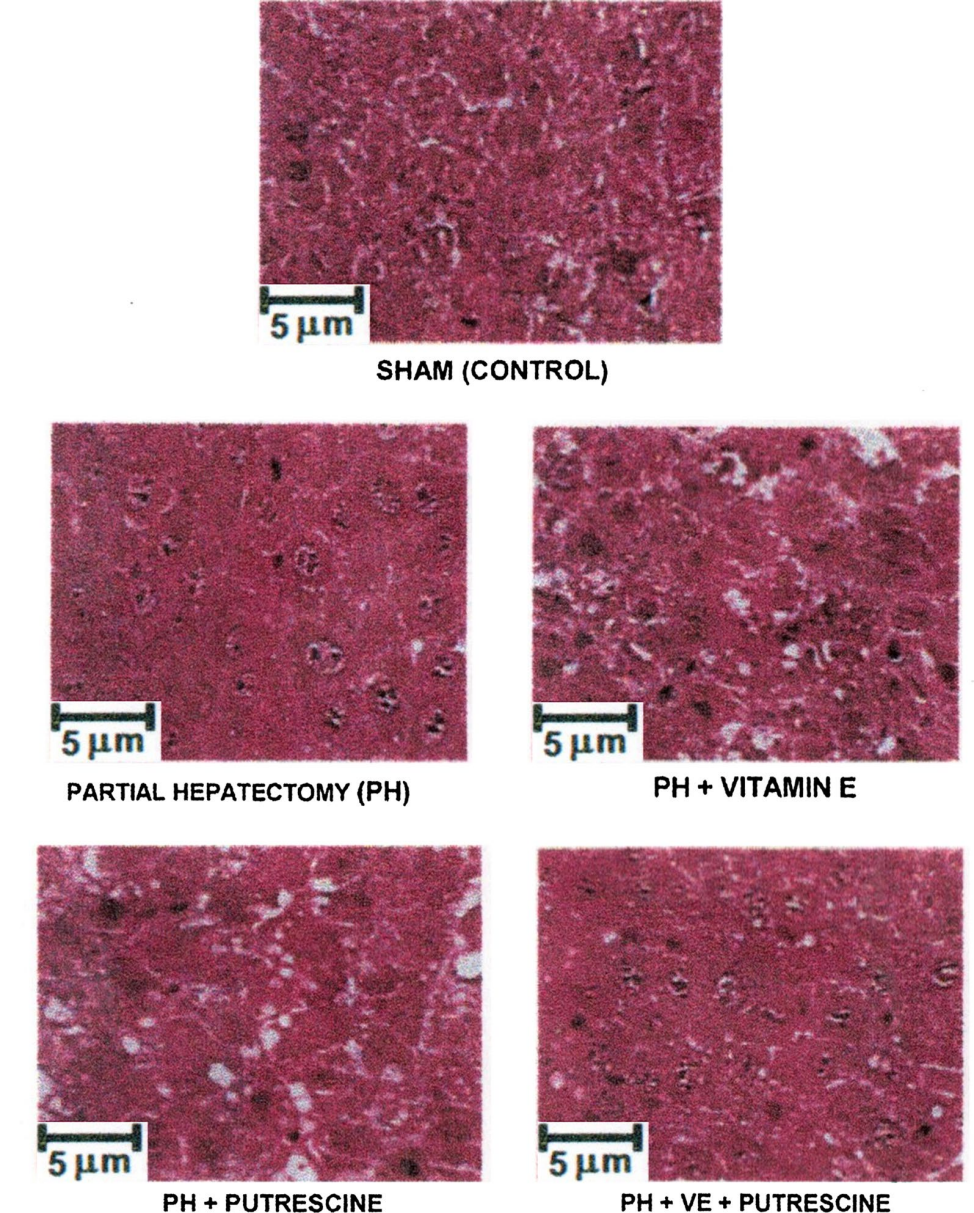
Liver histology and mitotic images in animals subjected to PH and treated with α-tocopherol and/or putrescine. Representative micrographs of liver (stained with hematoxylin and eosin) obtained from animals subjected to PH and treated with α-tocopherol and/or putrescine. Note the fatty infiltration and the representative mitotic images

The cytosolic activity of TK is considered as a reliable parameter for evaluating DNA synthesis [27]. The PH induced three peaks of TK activity (24–72 h after surgery; Fig. 1). Pre-treatment with α-tocopherol elicited only two smaller increases at 24 and 48 h after PH (Fig. 1). Administration of putrescine did diminish the latter peaks (48 and 72 h), whereas administration of putrescine to α-tocopherol-treated PH-rats only elicited a small peak of TK activity at 48 h post-PH (Fig. 1). After PH, livers showed abundant mitotic images, peaking at 48 post-PH; however, the rate of mitosis was reduced after α-tocopherol pre-treatment (Figs. 1, 2). Putrescine treatment showed similar number of mitotic cells that found in PH-animals only, but also reversed the effects of α-tocopherol, eliciting a mitotic pattern similar to that found in PH-animals only (Figs. 1, 2). Recovery of liver mass was significantly diminished with α-tocopherol treatment, and restituted by the combined treatment with putrescine (Fig. 1); in addition, livers showed a slight fatty infiltration accompanied by abundant mitotic images after PH. With α-tocopherol, fatty liver was more evident and mitotic index was reduced (Fig. 2). Treatment with putrescine to PH rats increased fatty infiltration, but maintained the number of mitotic cells (Fig. 2). The combined treatment reversed the α-tocopherol effects on fatty accumulation and on the liver mitotic index (Figs. 1, 2).

### Oxidant status in liver subcellular fractions in animals subjected to PH and treated with α-tocopherol and/or putrescine

**Fig. 3:**
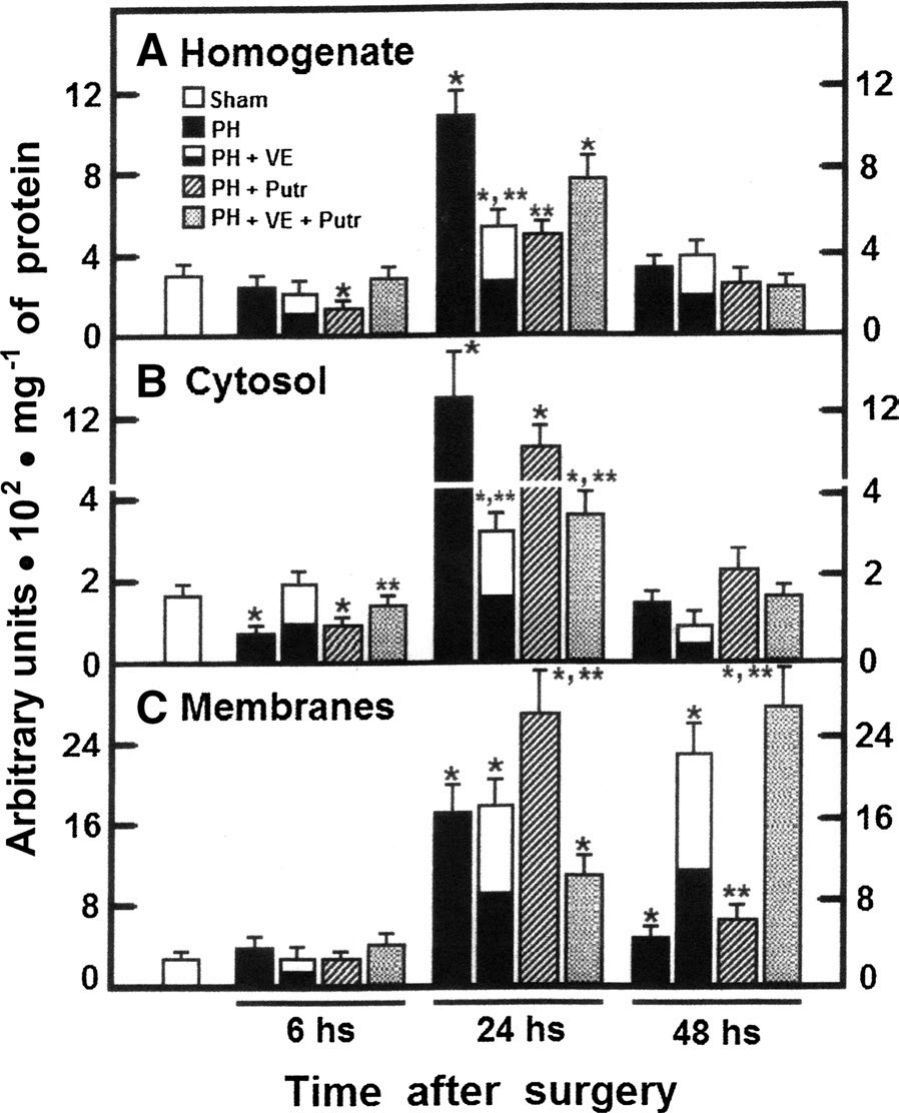
Generation of ROS by liver subcellular fractions obtained from animals subjected to PH and treated with α-tocopherol and/or putrescine. Results are the mean ± SE of arbitrary units of fluorescence (normalized) per mg^−1^ of protein for 5 individual observations per experimental point from total homogenate (*panel* **A**), cytoplasmic (*panel* **B**), and plasma membrane (*panel* **C**) fractions. *Symbols* for each experimental group are indicated at the *top* of the figure. Statistical significance as pointed out in Fig. 1

The PH promoted an increased ROS content in whole homogenate, at 24 h after surgery, that rapidly declined at 48 h post-PH, when compared with the control animals (Fig. 3A) [10, 11]; this increase was accounted for by the cytosol and plasma membranes fractions (Fig. 3B, C). Pre-treatment with α-tocopherol diminished mainly the cytosolic amount of 2,7-DCF fluorescent reactive products, without affecting its content in plasma membranes (Fig. 3c). Putrescine also diminished homogenate ROS, but elicited higher plasma membrane for 2,7-DCF fluorescent reactive products (24–48 h post-surgery). The combined treatment also augmented the levels of ROS, displaying the peak for 2,7-DCF fluorescent reactive products in plasma membranes from 24 to 48 h after surgery (Fig. 3C). Similar results in the pattern of LP by-products were obtained through assessing thiobarbituric acid reactive substances (TBARS), as previously reported [11].

### The ODC activity and its expression (ODC protein) in livers from animals subjected to PH and treated with α-tocopherol and/or putrescine

**Fig. 4:**
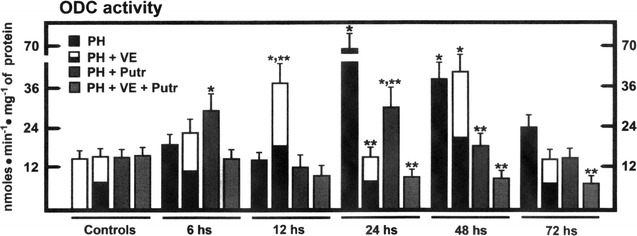
Liver ODC activity in the cytosolic fraction obtained from animals subjected to PH and treated with α-tocopherol and/or putrescine. Results are the mean ± SE of 5 individual observations per experimental group. *Symbols* for each experimental group are indicated at the *top* of the figure. Statistical significance as pointed out in Fig. 1

**Fig. 5:**
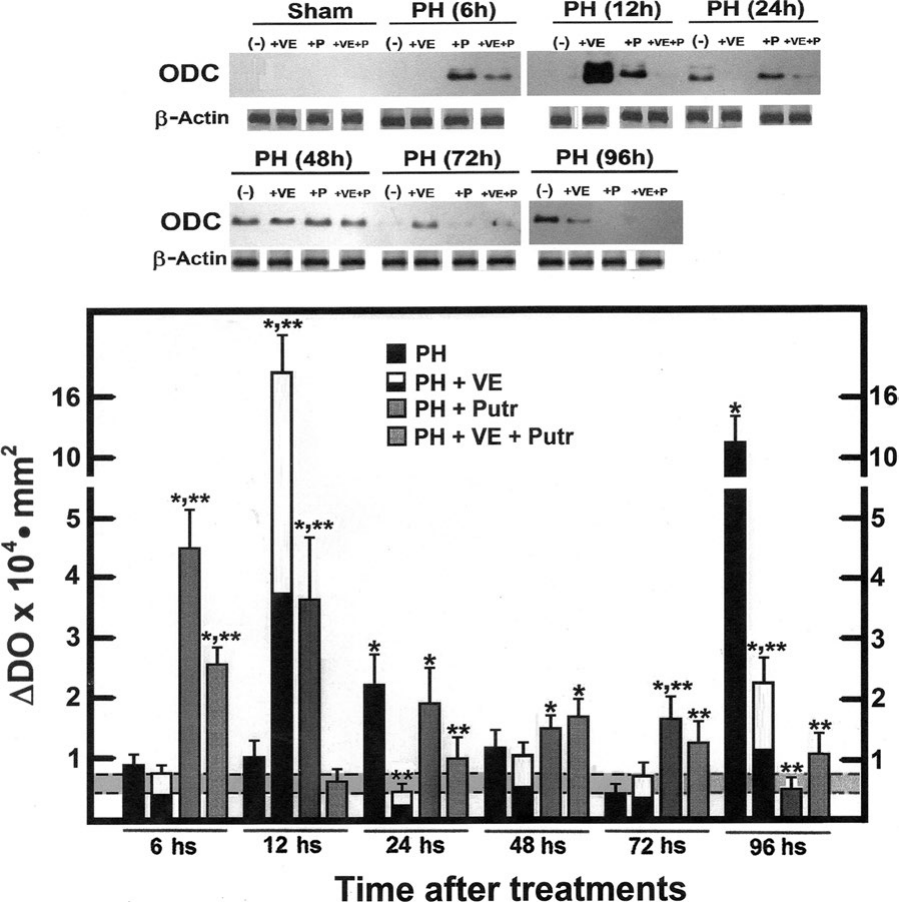
Liver ODC expression (ODC protein) detected in the cytosolic fraction obtained from animals subjected to PH and treated with α-tocopherol and/or putrescine. Results are the mean ± SE of 5 individual observations per experimental group. At the *top* of the figure, there is a representative western blot analysis for ODC in each experimental group and time; at the *bottom*, it is shown a densitometric analysis of the blots for ODC (Controls = *shadowed bar*). Protein load was corrected by a corresponding western blot for β-actin. *Symbols* for each experimental group are indicated at the *top* of the figure. Statistical significance as pointed out in Fig. 1

The PH induced two peaks of ODC activity (24 and 48 h after surgery; Fig. 4). Pre-treatment with α-tocopherol shifted earlier the increased ODC activity, without modifying the second peak (Fig. 4). Putrescine evoked an unexpected early increase of ODC activity (6 h), significantly reducing the PH-induced further increased ODC activity. The combined treatment blunted all increases in ODC activity elicited by either PH (Fig. 4). Increased ODC activity correlated well with enhanced ODC protein (at 24 h), whereas another peak was noted 96 h after surgery (Figs. 4, 5). The peak of ODC activity (12 h post-surgery) only correlated well with its expression at this time, and α-tocopherol also decreased the latter peak of ODC expression (Fig. 5). With putrescine treatment, ODC activity and expression only correlated well at 6 h, and ODC expression continued higher, thereafter (Fig. 5), without a concomitant increase in its activity (Fig. 4). Moreover, ODC expression was also increased early (6 h), followed by smaller increases in the content of ODC protein (24–96 h), in the group receiving both treatments (Fig. 5).

### Liver levels of polyamines in animals subjected to PH and treated with α-tocopherol and/or putrescine

**Fig. 6:**
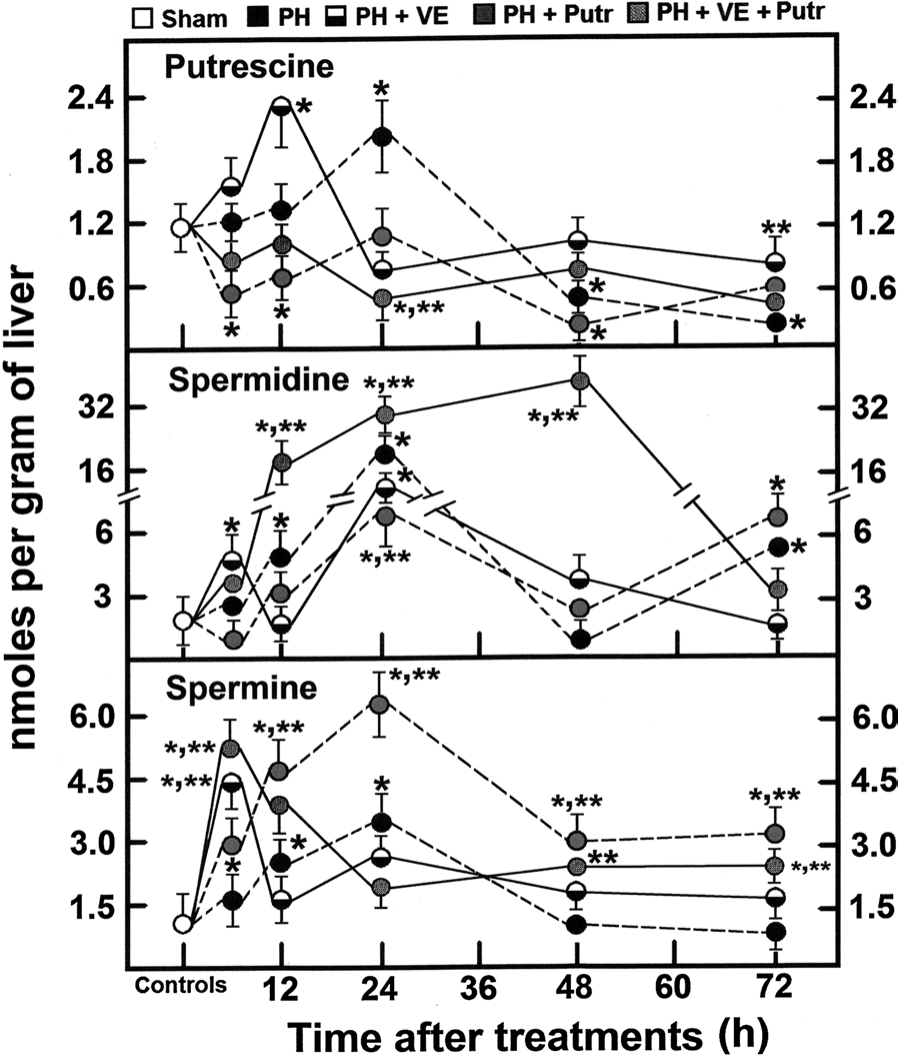
Liver levels of putrescine, spermidine, and spermine in animals subjected to PH and treated with α-tocopherol and/or putrescine. Results are the mean ± SE of 5 individual observations per experimental group. The levels for liver putrescine, spermidine, and spermine, detected through HPLC, are expressed as nmol g^−1^ of liver. *Symbols* for each experimental group are indicated at the *top* of the figure. Statistical significance as indicated in Fig. 1

The Fig. 6 shows the levels of the main polyamines (putrescine, spermidine, and spermine) after PH and under the different treatments. Starting at 6 h after PH, levels of the three polyamines (putrescine, spermidine and spermine) were gradually increased, reaching a maximal value at the first peak of DNA synthesis (24 h), and decreased at 48 h post-PH (Fig. 6). α-Tocopherol increased even earlier spermidine and mainly spermine at 6 h, while putrescine peaked at 12 h post-PH and spermidine at 24 h after surgery. Interestingly, polyamines rapidly dropped thereafter, reaching its lowest value at 72 h post-PH with the α-tocopherol treatment (Fig. 6). Putrescine treatment induced a rapid increase in spermidine and spermine levels (6–24 h), but levels for putrescine were decreased at 48 h (Fig. 6). Putrescine blocked the effects of α-tocopherol on polyamine metabolism during the first 12 h after PH; however, within 48–72 h post-surgery, putrescine seemed to be converted into spermidine and spermine, when compared with PH-rats treated with α-tocopherol only (Fig. 6).

### Liver levels of ornithine and citrulline in animals subjected to PH and treated with α-tocopherol and/or putrescine

The levels of liver ornithine, as the substrate for polyamine synthesis, were also measured. In the PH, liver ornithine levels tended to increase at 24 h after PH, but without a statistically significance. In turn, α-tocopherol treatment increased liver content of ornithine, except at 24 h post-PH, and putrescine alone showed an early increase for this amino acid (6 h), normalizing thereafter (Table 1). In the group of PH-animals with the combined treatments, the highest ornithine levels were obtained, when compared with controls rats (Table 1). A significant fraction of ornithine was converted to putrescine and spermidine, reaching the lowest ornithine/spermidine ratio after 24 h post-PH, which was now increased at 72 h post-surgery (Table 1). In PH-animals pre-treated with α-tocopherol, the ornithine/spermidine ratio was early increased (at 12 post-surgery), and “inverted” at 48–72 h post-PH (Table 1). Exogenous administration of putrescine to PH-animals elevated its ratio at all times tested, except at 72 h (Table 1). The combined treatment of α-tocopherol with putrescine greatly stimulated ornithine utilization for polyamine synthesis (12–48 h after surgery), decreasing the ornithine/spermidine ratio and apparently blocking the α-tocopherol effects in PH-animals.

**Table 1:** Liver levels of ornithine and citrulline in animals subjected to PH and treated with α-tocopherol and/or putrescine

Treatment	Ornithine	Citrulline	Ornithine/citrulline ratio
Sham (control)	378 ± 57	529 ± 63	0.71 ± 0.09
PH + vehicle			
6 h post-PH	257 ± 26	438 ± 53	0.59 ± 0.06
12 h post-PH	345 ± 41	575 ± 81	0.60 ± 0.07
24 h post-PH	551 ± 77	631 ± 101	0.87 ± 0.12
48 h post-PH	515 ± 67	746 ± 104	0.69 ± 0.10
72 h post-PH	458 ± 51	309 ± 37*	1.48 ± 0.23*
PH + VE			
6 h post-PH	513 ± 62**	616 ± 80	0.83 ± 0.10
12 h post-PH	761 ± 107*^,^**	614 ± 92	1.24 ± 0.18*^,^**
24 h post-PH	569 ± 91	584 ± 99	0.97 ± 0.16
48 h post-PH	608 ± 85**	507 ± 76	1.20 ± 0.17*^,^**
72 h post-PH	746 ± 90*^,^**	415 ± 54	1.80 ± 0.23*^,^**
PH + Putrescine			
6 h post-PH	468 ± 80**	440 ± 52	1.06 ± 0.15**
12 h post-PH	510 ± 77	185 ± 24*^,^**	2.76 ± 0.39*^,^**
24 h post-PH	548 ± 71	601 ± 90	0.91 ± 0.13
48 h post-PH	705 ± 78*	805 ± 137	0.88 ± 0.12
72 h post-PH	488 ± 44	303 ± 39*	1.48 ± 0.16*
PH + VE + Putres			
6 h post-PH	983 ± 118*^,^**	898 ± 135**	1.04 ± 0.14**
12 h post-PH	587 ± 82**	257 ± 41*^,^**	2.28 ± 0.34*^,^**
24 h post-PH	674 ± 106*	690 ± 97	0.98 ± 0.15
48 h post-PH	755 ± 78*^,^**	938 ± 131*	0.80 ± 0.10
72 h post-PH	535 ± 55	217 ± 27*	2.47 ± 0.28*^,^**

Results are the mean ± SE of 5 individual observations by experimental point and expressed by gram of liver

*PH* partial hepatectomy, *VE* α-tocopherol, and *Putres* putrescine. Statistical significance, as indicated in *

The amount of citrulline, another ornithine by-product (urea cycle), was decreased at later times post-PH, diminishing the ornithine/citrulline ratio, indicative for a diminished citrulline synthesis (Table 1). In PH-rats pre-treated with α-tocopherol, ornithine predominated over citrulline, probably by a diminution of urea production, while putrescine alone promoted increased values for the ornithine/citrulline ratio (Table 1). In the group of the combined treatment, variations in citrulline levels gave ornithine/citrulline ratios similar to those found in the PH group treated with putrescine only (Table 1).

### Liver amount of retinoids in livers from animals subjected to PH and treated with α-tocopherol and/or putrescine

**Fig. 7:**
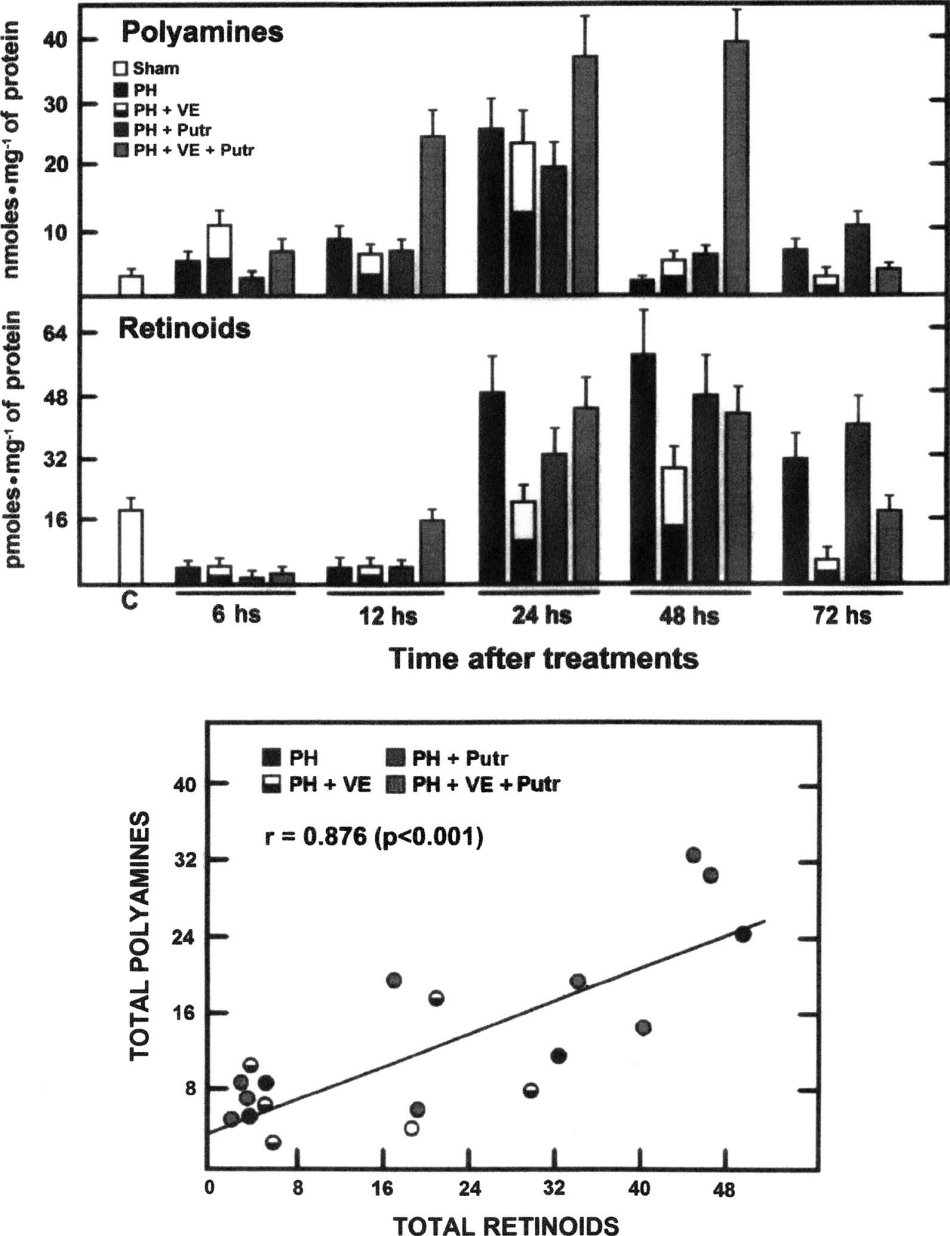
Liver levels of total polyamines, retinoids and its correlation in animals subjected to PH and treated with α-tocopherol and/or putrescine. Results are the mean ± SE of 5 individual observations per experimental group. *Upper*, total polyamines (expressed as nmol mg^−1^ of protein) is the sum of putrescine, spermidine, and spermine shown in Fig. 6, while retinoid levels (expressed as pmol mg^−1^ of protein) corresponded to the sum of retinol, retinal, and retinoic acid. *Bottom*, a linear regression analysis provided a significant straight correlation (p < 0.001) between fluctuations of polyamines and those of retinoids, with a high correlation coefficient (r = 0.876). *Symbols* for each experimental group are indicated at the *top* of the figure

Figure 7 shows the levels of the main retinoids (retinol, retinal, and retinoic acid), as well as those of total polyamines. Early after PH (6–12 h post-surgery), the retinoid levels were decreased when compared with sham-operated controls, and either α-tocopherol or putrescine did not modify this pattern (Fig. 7). At the peak of DNA synthesis (24 h), retinoid levels increased in the experimental groups, except in PH-rats pre-treated with α-tocopherol; however, at 72 h post-PH, animals subjected to PH and pre-treated with α-tocopherol had significantly lower retinoid concentrations, effect that was normalized after administering putrescine (Fig. 7). From here, we found a very significant correlation between both retinoid and polyamines levels (r = 0.876, p < 0.001) throughout the progression of liver regeneration (Fig. 7).

## Discussion

In the present study, the α-tocopherol-induced inhibition of PH-induced rat liver regeneration was accompanied by altered amount of ODC, its activity, and the temporality of polyamine production. Pre-treatment with α-tocopherol delayed for 24 h the LP peak in the plasma membranes (Fig. 3), and induced a fatty liver and a decreased number of mitotic images in hepatocytes (Figs. 1, 2), decreasing the rate of cell proliferation (Fig. 1). In addition, α-tocopherol promoted a drastic overexpression of ODC at early times post-PH (Fig. 3), correlating with a desynchronized production of spermidine and spermine (Fig. 6), and also an altered ornithine metabolism (Table 1). ROS by-products becomes relevant in the metabolic adjustment of the proliferating liver [31], and we have shown that LP plays a role during the progression of rat liver proliferation [8], probably initiating a general cell response [32]. Indeed, liver metabolism of retinoids, influenced by the NAD+ -dependent ADH activity and cell redox state, are important for the progression of rat liver regeneration, through the expression of STAT proteins [11].

As to polyamine metabolism, ROS and nitrogen species inactivate methionine adenosyltransferase I/III [33], reducing hepatic S-adenosylmethionine levels, which can regulate liver regeneration by forming spermidine and spermine [34, 35]. Furthermore, in colon carcinoma cells (HT-29 cells), the altered ornithine (ODC substrate) flux through urea cycle can lead to ammonia accumulation, reducing ODC activity which results in a decreased polyamine synthesis [36], indicating that the metabolic fate of ornithine is also involved in the polyamine synthesis.

The overexpression of ODC increases putrescine pool [37], and transgenic rats with conditioned spermidine/spermine N^1^-acetyltransferase expression, fail to initiate liver regeneration [38]. From here, synchrony in polyamine synthesis appears to be essential to drive an adequate liver cell proliferation; interestingly, administration of putrescine in PH rats pre-treated with α-tocopherol restored the mitotic rate in the regenerating liver. Since putrescine administered to PH animals improves DNA synthesis in the rat regenerating liver under either, pro-oxidant (ethanol treatment; ref. [19]) or antioxidant conditions (α-tocopherol administration), this would suggest an involvement of cellular oxidative status in the control of polyamine metabolism.

The inhibitory effects of α-tocopherol on PH-induced rat liver regeneration seem to be due to a kind of modulation of cell signaling pathways [3]. The α-tocopherol-induced early ODC overexpression and its corresponding activity (Figs. 4, 5) could partially block subsequently adaptive step required for PH-induced regeneration. Indeed, ODC expression and activity does not always correlate well, suggesting that ODC could be regulated at the post-translational level [19], including a non-covalent binding to an inhibitory “antizyme”, microsomal oxidation, transglutamination, and phosphorylation [39, 40]. It has been suggested that hepatic putrescine content only can be essential for liver regeneration after PH [41]. However, the opposite has been also reported, suggesting that spermidine and/or spermine, but apparently not putrescine, are required for liver regeneration [38]. A relationship between rat liver regeneration and the concentration ratio of spermidine/spermine [33]; in the present study, we also noted that ornithine/putrescine and ornithine/citrulline could be also important (Table 1). These ratios indicate that liver ornithine metabolism through ornithine carbamoyltransferase provides an alternative metabolic pathway for ornithine (urea cycle), competing for this substrate [42].

It is difficult to explain the restituting effects of putrescine in the mitosis index from livers obtained from PH-animals pre-treated with the VE, since the concomitant treatment did not increase TK and ODC activities, nor expression of the ODC protein (Figs. 1, 3, 4). As to the group of rats subjected to PH and receiving putrescine, this polyamine did favor a higher generation of LP by-products in plasma membranes (24 h; Fig. 3). This suggests that putrescine readily modified by itself the progression of PH-induced liver regeneration, mainly through shifting earlier the peak for ODC activity (Figs. 4, 5). Therefore, results suggest that PH-induced cytosolic changes in LP by-products levels, can be driven to produce enough content of putrescine, for its further metabolism. In this regard, nuclear oxidation of spermine could increase the production of highly reactive H_2_O_2_, having an increased potential for oxidative DNA damage in cancerous cells [43, 44]. In fact, polyamines also have a role in facilitating cell death, and the ability of polyamines to alter DNA–protein and protein–protein interactions might be disruptive to cellular functions, which indicates that polyamine pathway can be a molecular target for therapeutic intervention in several types cancers [45].

Interestingly, the balance in antagonistic activities of ODC and SSAT in the stressed hepatoma cells resulted in increased cell polyamine content. The catabolism of polyamines by SSAT generates toxic products that promote carcinogenesis, whereas polyamine synthesis is favorable for proliferation of cells [46]. However, it is not clear why putrescine induced an enhanced ODC expression; possibly an increased putrescine availability by its exogenous administration might modify the ODC turnover, which is known to be rapid.

The aldehyde dehydrogenase 1a2 (RALDH2), which is the rate-limiting enzyme in the production of retinoic acid from retinaldehyde, and highly induced in the regenerating heart, gives evidence that retinoic acid plays a key role [47]. Retinoids can decrease expression of Bcl-2, and the combination of 13-cis retinoic acid and interferon enhanced the effect of paclitaxel chemotherapy, resulting in that this combination can be safely administered in phase I studies [48]. Interactions between polyamines and retinoids can regulate retinoid-induced apoptosis in Jurkat cells [23]; similarly, retinoic acid activates transglutaminase that conjugates with putrescine, probably leading to a simultaneous inhibition of DNA synthesis in PH-animals [49, 50]. Polyamines are positively charged organic cations that can physiologically interact with macromolecules such as DNA and RNA. Taking advantage of this property, natural and synthetic polyamines could be used as polyamine-substituted agents carried out for non-viral gene delivery vehicles for therapeutic purposes [51]. In fact, modeling of polyamine-protein conjugates shows that this conjugation induces major alterations of serum protein conformations, and that polyamine-protein interaction is spontaneous and chitosan nanoparticles can be used for delivery of antitumor polyamine analogues [52, 53].

Here, the α-tocopherol effects on rat liver regeneration beyond 24 h could highlight the relevance of early changes in the oxidative status of the PH-induced rat proliferating liver, since VE administration might induce a ‘premature’ but ineffective proliferative response. What could be the mechanism(s) underlying the present findings? After PH, there is a transient increase of ROS mainly in plasma membranes and cytoplasm [8]. The latter (cytosolic fraction) could be part of a signaling pathway activating STATs translocation into the nucleus, as well as stimulating cyclin D1 expression [10, 11]. In fact, we have demonstrated that increased serum levels of cytoplasmic enzymes observed during PH-induced rat liver regeneration is differentially regulated by modifications of the oxidant status, indicating that this release is a strictly controlled event [54].

Therefore, it is possible that α-tocopherol makes earlier the adaptive changes induced by PH and partially blocks liver cell proliferation, which is largely ameliorated by putrescine, restituting in synthesis of retinoids (Fig. 7) and “normalizing” progression of rat liver regeneration, apparently returning the synchrony of polyamines and retinoid metabolisms. Indeed, our results suggest that the main effect of VE was of “desynchronizing” (shifting earlier) rather than to partial block polyamine synthesis and metabolism by the PH-induced rat regenerating liver. In fact, uncontrolled oxidative stress is involved in the hypocontractility of visceral artery to vasoconstrictors and formation of hyperdynamic circulation in cirrhosis with portal hypertension [55]. This stress the role of a selective control of ROS and LP by-products in liver regeneration, probably mediated by variations in STATs expression, as well as those on polyamine and retinoid metabolism.

## Conclusions

The pretreatment with α-tocopherol was capable to shift early the increased activities found for TK and ODC, inducing a dramatic overexpression for the ODC protein. These effects seemed to be related to the antioxidant action exerted by α-tocopherol and occurring during inhibition of PH-induced rat liver regeneration. Polyamine synthesis and catabolism were also temporally affected by pre-treatment with α-tocopherol. Although administration of putrescine induced minor changes in the liver of animals subjected to PH, we noted that the concomitant treatment actually counteracted most adverse actions exerted by α-tocopherol on rat liver regeneration, restoring the proliferative potential in the remnant liver and the levels of liver retinoids, apparently restituting this “synchrony” between both metabolism. These results could represent a novel mechanism underlying the inhibitory action of VE on rat liver regeneration, which can be implicated in pathologies occurring with liver cell proliferation, such as cirrhosis and hepatocarcinoma, and contribute to the ongoing design of possible new therapeutic interventions.

## Abbreviations

ADH: alcohol dehydrogenase
H2DCF-DA: 2′,7′-dichlorodihydrofluorescein di-acetate
ODC: ornithine decarboxylase
PCNA: proliferating cell nuclear antigen
PH: partial hepatectomy
Putres: putrescine
ROS: reactive oxygen species
Sperd: spermidine
Sperm: spermine
STAT: signal transducer and activator of transcription
TK: thymidine kinase
VE: vitamin E
